# Mapping Sanfilippo Syndrome: A Multisystem Clinicopathological Autopsy

**DOI:** 10.3390/diagnostics16101527

**Published:** 2026-05-18

**Authors:** Mioara-Florentina Trandafirescu, Elena-Roxana Avădănei, Nina Filip, Catalina Iulia Saveanu, Iolanda Foia, Vasilica Toma, Livia Genoveva Baroi, Dana-Teodora Anton-Paduraru, Stefana Maria Moisa, Ludmila Lozneanu

**Affiliations:** “Grigore T. Popa” University of Medicine and Pharmacy, 16 University Street, 700115 Iasi, Romania; mio.trandafirescu@umfiasi.ro (M.-F.T.); nina.zamosteanu@umfiasi.ro (N.F.); catalina.saveanu@umfiasi.ro (C.I.S.); iolanda.foia@umfiasi.ro (I.F.); vasilica.toma@umfiasi.ro (V.T.); livia.baroi@umfiasi.ro (L.G.B.); dana.anton@umfiasi.ro (D.-T.A.-P.); stefana-maria.moisa@umfiasi.ro (S.M.M.); ludmila.lozneanu@umfiasi.ro (L.L.)

**Keywords:** mucopolysaccharidosis type III, Sanfilippo syndrome, lysosomal storage disorder, autopsy, histopathology, glycosaminoglycans, foam cells, multisystem involvement

## Abstract

**Background/Objectives**: Mucopolysaccharidosis type III (MPS III, Sanfilippo syndrome) is an autosomal recessive lysosomal storage disorder caused by deficiencies in enzymes required for heparan sulfate degradation. While primarily recognized for its devastating neurodegenerative course, the systemic extent of glycosaminoglycan (GAG) accumulation remains under-characterized. This study aims to provide a detailed multisystemic pathological mapping of MPS III to challenge the traditional “brain-only” disease paradigm and highlight the clinical relevance of extracerebral involvement. **Methods**: We present a comprehensive clinicopathological analysis of a 15-year-old female patient with a history of profound neuropsychomotor delay, refractory epilepsy, and spastic tetraplegia. Following her death due to terminal bronchopneumonia during palliative care, a complete forensic and pathological autopsy was conducted. Tissue samples from all major organ systems were processed using routine Hematoxylin–Eosin (HE) staining, immunohistochemical staining for CD68, and specialized histochemical stains to identify intracellular storage products. **Results**: Macroscopic evaluation revealed significant diffuse cerebral atrophy, meningoencephalic edema, cardiac valvulopathy with compensatory myocardial remodeling, and hepatosplenomegaly. Furthermore, erosive gastrointestinal lesions and degenerative renal changes were identified. Histopathological examination confirmed widespread cytoplasmic vacuolization across diverse cell populations, including neurons, hepatocytes, renal tubular cells, and the reticuloendothelial system. These findings demonstrate that GAG deposition is a generalized process affecting nearly every parenchymal structure. **Conclusions**: Although neurological decline dominates the clinical phenotype, our findings underscore that MPS III is a true systemic storage disorder. Significant involvement of the cardiovascular and visceral systems contributes to the disease’s complexity and mortality. This case reinforces the critical diagnostic value of a comprehensive autopsy in delineating the full morphological spectrum of Sanfilippo syndrome, providing essential insights for multidisciplinary management.

## 1. Introduction

Mucopolysaccharidoses constitute a heterogeneous group of inherited metabolic disorders caused by deficiencies of lysosomal enzymes responsible for the degradation of glycosaminoglycans [1,2]. MPS III (mucopolysaccharidosis type III) results from impaired catabolism of heparan sulfate due to mutations affecting one of several specific enzymes, leading to gradual intralysosomal substrate accumulation and cellular dysfunction [3,4]. Each of these enzymes determine a different type of MPS III (IIIA, IIIB, IIIC, IIID and IIIE), as follows: heparan N-sulphatase (also known as sulfamidase) (SGSH) in MPS IIIA, α-N-acetylglucosaminidase (NAGLU) in MPS IIIB, heparan acetyl CoA: α-glucosaminide N-acetyltransferase (HGSNAT) in MPS IIIC, N-acetyl glucosamine sulfatase (GNS) in MPS IIID, and N-glucosamine 3-O-sulfatase (arylsulfatase G or ARSG) in MPS IIIE. The fifth proposed subtype, MPS IIIE, appears to exhibit a clinical phenotype distinct from the classic forms of MPS III [5].

Lysosomal impairment disrupts cellular homeostasis, promotes chronic neuroinflammation, and triggers ongoing neuronal loss [2,6,7]. Clinically, patients typically present with developmental delay followed by cognitive regression, behavioral disturbances, epilepsy, and progressive motor deterioration [8,9,10]. Diagnostic delay and misdiagnosis of idiopathic developmental delay, ADHD, or Autism Spectrum Disorder (ASD) are frequently reported [9,11]. Although the central nervous system is considered the primary target, increasing evidence suggests that systemic somatic involvement may be underrecognized and insufficiently characterized at the morphopathological level [12,13,14].

Unlike MPS types I and II, where somatic manifestations are clinically dominant [15,16], MPS III is traditionally characterized as a primary neurodegenerative condition with more subtle visceral features [17].

Therapeutic limitations in mucopolysaccharidosis type III result from the inability of conventional enzyme replacement therapies to cross the blood–brain barrier, thereby restricting central nervous system efficacy and maintaining the predominantly neurodegenerative course of the disease. However, imaging and laboratory investigations frequently demonstrate hepatosplenomegaly, cardiac abnormalities, and progressive multisystem changes [12,18,19,20], indicating broader organ involvement. Although overall survival is largely determined by progressive neurological decline, quality of life and acute clinical complications are frequently influenced by systemic dysfunction, particularly cardiac and respiratory involvement [21,22].

In this context, post mortem examination remains a crucial tool for accurately assessing disease burden and correlating clinical manifestations with their histopathological substrate [2].

The aim of the present study was to provide a detailed macroscopic and microscopic characterization of organ involvement in an advanced case of MPS III, emphasizing the systemic impact of this disorder.

## 2. Materials and Methods

A complete autopsy was performed according to standard protocol. The study was conducted in accordance with the Declaration of Helsinki and approved by the Ethics Committee of Children’s Emergency Hospital “Sf. Maria” in Iași (March 2026). Written informed consent was obtained from the patient’s legal guardians for the publication of this case report and any accompanying images.

Major organs were systematically examined macroscopically, and representative tissue samples including the central nervous system, heart, lungs, liver, spleen, kidneys, pancreas, gastrointestinal tract, and aorta were harvested. These samples were fixed in 10% buffered formalin, paraffin-embedded, and sectioned at 4–5 μm.

For histopathological analysis, routine Hematoxylin–Eosin (H&E) staining, Masson’s trichrome stain, Sharlach stain and periodic acid–Schiff (PAS) stain were used. Three independent pathologists (M.F.T., E.R.A. and L.L.) examined the histological slides and identified the microscopic lesions.

Additionally, lipid accumulation within subendothelial foamy macrophages of the aorta was evaluated using Scharlach staining and the neuroinflammatory landscape was further characterized using immunohistochemistry for CD68 on selected sections.

The definitive diagnosis of lysosomal storage was established through a multidisciplinary approach, integrating clinical history, biochemical findings, and classical histomorphological features. However, it should be noted that immunohistochemical (IHC) analysis for specific lysosomal membrane proteins, such as LAMP1, LAMP2, or LIMP2, was not performed due to institutional resource constraints. Despite the absence of these specific IHC markers, the correlation between the established biochemical deficit and the characteristic microscopic findings (e.g., diffuse vacuolization and PAS-positivity) was considered sufficient to characterize the systemic pathological involvement in this advanced stage of MPS IIIA.

## 3. Results

### 3.1. Case Presentation

A 15-year-old female patient with advanced neurodegenerative encephalopathy secondary to mucopolysaccharidosis type IIIA (MPS IIIA) was admitted to the palliative care unit of the Children’s Emergency Hospital “Sf. Maria” in Iași by inter-hospital transfer from a tertiary medical center in another city due to progressive clinical deterioration. The patient had been institutionalized in a residential care facility, where she had been institutionalized for several years because of her complex medical and social needs.

The diagnosis of MPS IIIA had been established in early childhood, at approximately 3 years of age, following developmental regression and behavioral disturbances. Biochemical investigations revealed increased urinary glycosaminoglycan excretion with predominance of heparan sulfate (often, over 10 µg/mg creatinine), and enzymatic assays confirmed the specific lysosomal deficiency.

Although genetic testing was not performed, the diagnosis of MPS IIIA was confirmed by the pathognomonic triad of severe clinical neurological deterioration, significantly elevated urinary glycosaminoglycans, and a documented deficiency in heparan-N-sulfatase activity during the enzymatic assay.

Her clinical course followed the typical advancing neurodegenerative trajectory of Sanfilippo syndrome. Early manifestations included language delay, cognitive stagnation, marked hyperactivity, behavioral dysregulation, and significant sleep disturbances characterized by fragmented sleep and nocturnal agitation. During mid-childhood, she developed progressive loss of previously acquired skills, worsening intellectual disability, and refractory epilepsy or drug-resistant epilepsy. Motor impairment gradually ensued, with increasing spasticity, gait instability, and eventual loss of independent ambulation, progressing toward severe spastic tetraplegia. Bulbar dysfunction with dysphagia and recurrent aspiration episodes led to the imperative usage of enteral nutritional support via percutaneous endoscopic gastrostomy.

By adolescence, the patient reached a terminal stage, characterized by a non-verbal state, bedridden status, and total dependence on attentive care in order to survive. These clinical manifestations reflect the relentless systemic accumulation of GAGs, leading to the extensive multisystem involvement subsequently documented during post mortem examination.

### 3.2. Macroscopic Findings

The patient showed marked malnutrition with generalized muscle wasting and severe osteoarticular contractures. External examination revealed synophrys (confluent eyebrows) and coarse hair texture, phenotypic features compatible with the dysmorphic characteristics described in certain lysosomal storage disorders.

**Figure 1:**
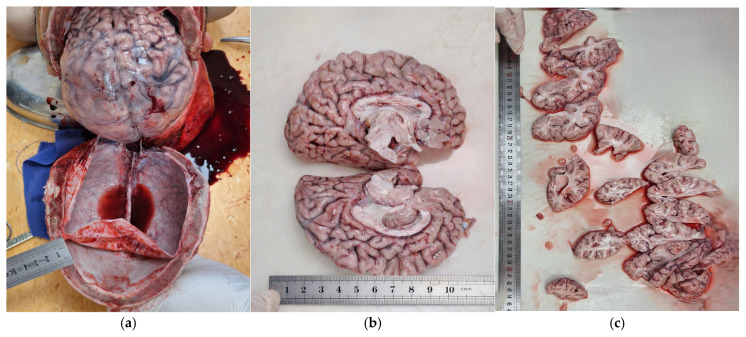
Gross appearance of the brain: (**a**) thickness of the cranial vault, cerebral edema, and vascular congestion, (**b**) ventricular dilatation and cortical thinning (sagittal slices), (**c**) parenchymal changes associated with advanced neurodegeneration (coronal slices).

The cranial vault thickness measured approximately 10 mm, markedly exceeding the expected reference range for a 15-year-old adolescent (approximately 5–6 mm), suggesting skull remodeling associated with lysosomal storage disease (Figure 1a).

The brain showed marked encephalomalacia or global cerebral atrophy with widened sulci, meningeal congestion, cerebral edema, and ventricular dilatation, reflecting advanced neurodegeneration. At post mortem analysis, the brain weighed 910 g, substantially below the normal reference range for a 15-year-old adolescent female (approximately 1200–1350 g), reflecting terminal-stage neurodegeneration (Figure 1b,c).

Macroscopic examination of the respiratory tract revealed mucosal congestion and edema. No structural abnormalities of the tracheal cartilage or evidence of tracheomalacia were identified. The lungs, however, showed multifocal consolidation consistent with terminal bronchopneumonia.

**Figure 2:**
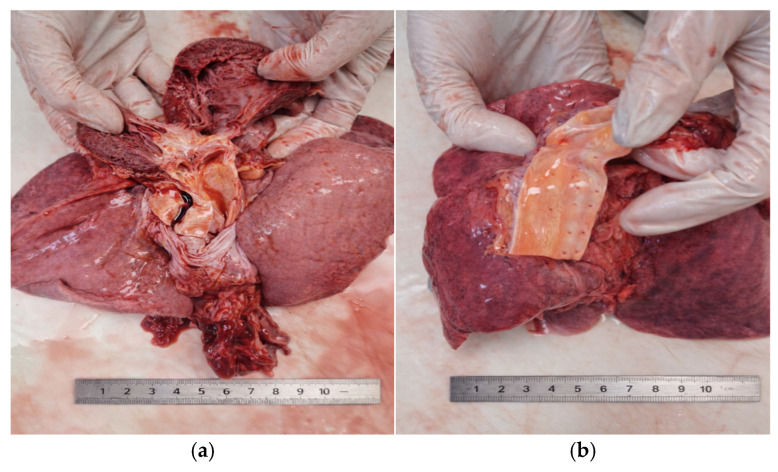
Macroscopic cardiac and vascular findings: (**a**) cardiomegaly, left ventricular hypertrophy, fibrous thickening of the mitral and aortic valves, (**b**) early lipid streaks (ascending aorta).

The heart displayed cardiomegaly secondary to left ventricular hypertrophy and fibrous thickening of the mitral and aortic valves. The heart weight was 450 g, markedly exceeding the expected normal range for a 15-year-old adolescent (approximately 220–250 g). This enlargement was characterized by left ventricular hypertrophy and diffuse fibrous thickening of both the mitral and aortic valves. The myocardium was pale and soft upon sectioning, consistent with chronic degenerative changes (Figure 2a).

In the ascending aorta, the intimal surface exhibited multiple longitudinal yellow streaks “fatty streaks”, indicating early lipid deposition within the subendothelial layer (Figure 2b).

**Figure 3:**
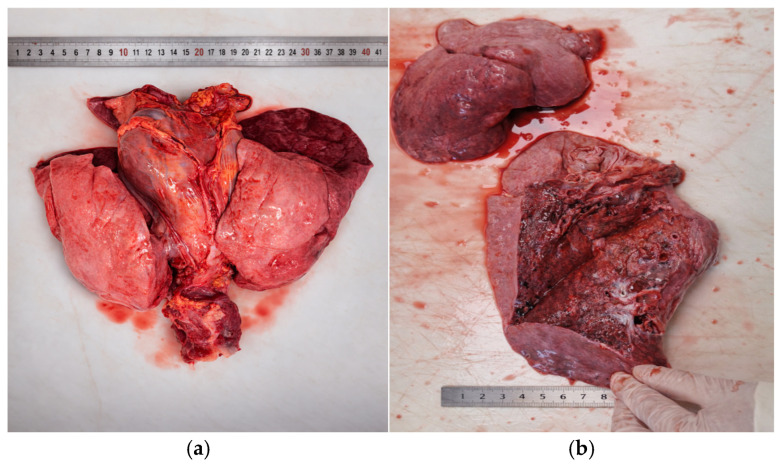
Gross appearance of the lungs: (**a**) congestion and dark-red pleural discoloration, (**b**) serohemorrhagic foamy fluid, with multifocal inflammatory consolidation.

The lungs were increased in weight (total approximately 950 g; reference range for age: 750–900 g), and appeared heavy, congested, and edematous, releasing serohemorrhagic foamy fluid on cut surface, with multifocal inflammatory consolidation consistent with bronchopneumonia (Figure 3a,b).

**Figure 4:**
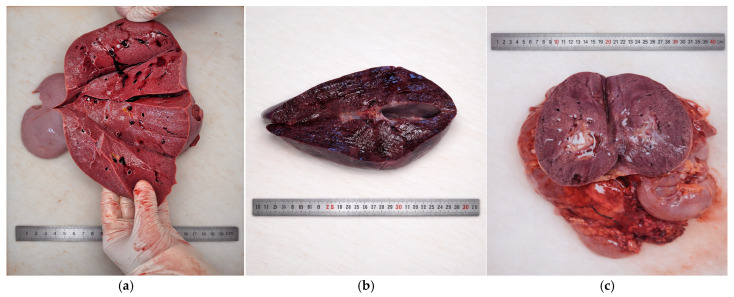
Macroscopic examination of abdominal organs: (**a**) liver showing diffuse dark-red congestion and parenchymal alterations, (**b**) spleen displaying enlargement and congestive appearance, (**c**) kidney with dark cortical discoloration and congestive parenchyma.

The liver was moderately enlarged and weighed approximately 1600 g (normal range for age: 1200–1400 g), with firm consistency and homogeneous appearance (Figure 4a), while the spleen demonstrated moderate splenomegaly, with a weight of approximately 220 g (normal range for age: 120–150 g), indicating systemic lysosomal storage disease (Figure 4b).

The kidneys demonstrated diffuse parenchymal congestion and mild interstitial edema, although corticomedullary differentiation remained preserved, weighing approximately 160 g (right) and 170 g (left) (reference range for age: 110–130 g) (Figure 4c).

The gastrointestinal tract exhibited erosive inflammatory lesions involving the gastroesophageal and intestinal mucosa. The gastrostomy site showed no macroscopic complications and no signs of local inflammation. Overall, the macroscopic findings were consistent with advanced multisystem metabolic disease with terminal cardiorespiratory decompensation. The mesenteric lymph nodes were enlarged due to the accumulation of macrophages containing stored GAGs, with maximum diameters ranging from 2 to 4 cm.

### 3.3. Microscopic Findings

Histological examination revealed diffuse cytoplasmic vacuolization affecting multiple tissues, characterised by lysosomal storage disorders.

**Figure 5:**
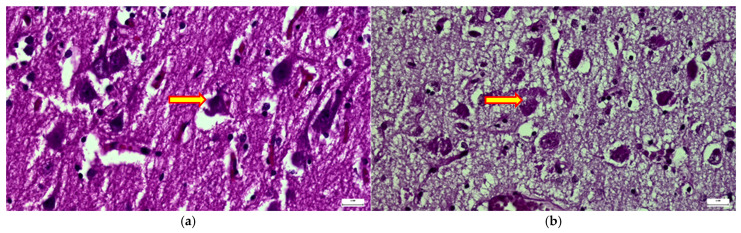

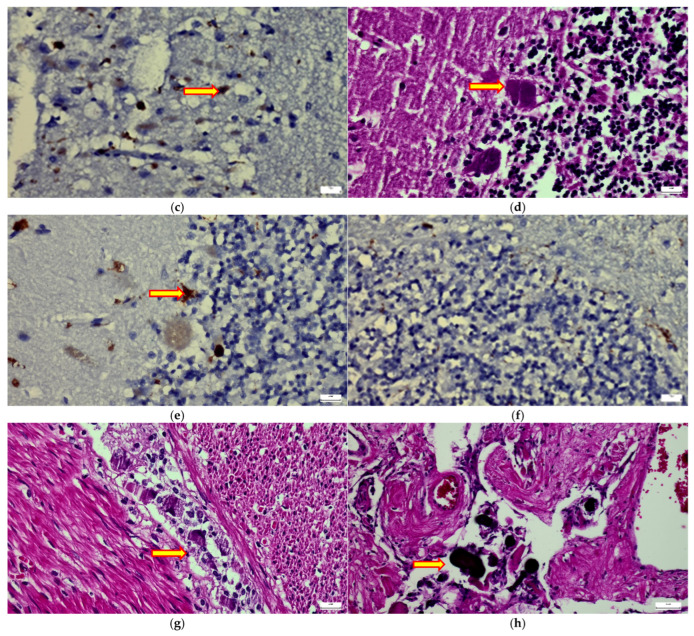
Histopathological analysis of the nervous system. (**a**,**b**) neocortical neurons and glial cells displaying diffuse cytoplasmic vacuolization and PAS-positive inclusions (HE and PAS, 20 µm). (**c**) perivascular distribution of CD68+ activated macrophages in the cerebral cortex (20 µm). (**d**) cerebellar Purkinje cells showing marked somatic ballooning and degenerative changes (HE, 20 µm). (**e**,**f**) comparative IHC for CD68 showing extensive microglial activation in the patient versus minimal reactivity in a healthy control (20 µm). (**g**) myenteric (Auerbach) plexus neuron with prominent vacuolization and “ballooning” appearance (HE, 20 µm). (**h**) choroid plexus showing stromal microcalcifications and vascular congestion (HE, 50 µm).

Within the central nervous system, neurons and glial cells showed prominent cytoplasmic vacuolization, interstitial edema, and early leukoencephalopathic changes, the latter representing a morphological indicator of advanced neurodegeneration (Figure 5a,b).

In the cerebellum, cortical and subcortical neurons show marked cytoplasmic ballooning and fine-to-coalescent vacuolization, consistent with intralysosomal accumulation of glycosaminoglycans (predominantly heparan sulfate). These changes are accompanied by neuropil rarefaction with a mild spongiform appearance, focal neuronal loss, microglial activation, and reactive gliosis.

Enlarged Purkinje cells have pale, vacuolated cytoplasm and degenerative changes, associated with decreased neuronal density, granular layer disorganization, and gliosis, reflecting progressive lysosomal storage-related neurodegeneration (Figure 5d).

In the colon in the myenteric (Auerbach) plexus, enteric ganglion neurons display prominent somatic distension and extensive cytoplasmic vacuolization (“ballooning degeneration”), occasionally with reduced neuronal density and perineural glial reaction, indicating involvement of the peripheral autonomic nervous system (Figure 5g).

Microcalcifications were also present in the brain, cerebellum, and predominantly perivascular choroid plexuses (Figure 5h).

Overall, the combined features demonstrate multisystem cardiac involvement in MPS III, characterized by intracellular storage in cardiomyocytes together with progressive interstitial fibrosis, contributing to myocardial stiffening, impaired contractility, and long-term cardiomyopathic changes.

**Figure 6:**
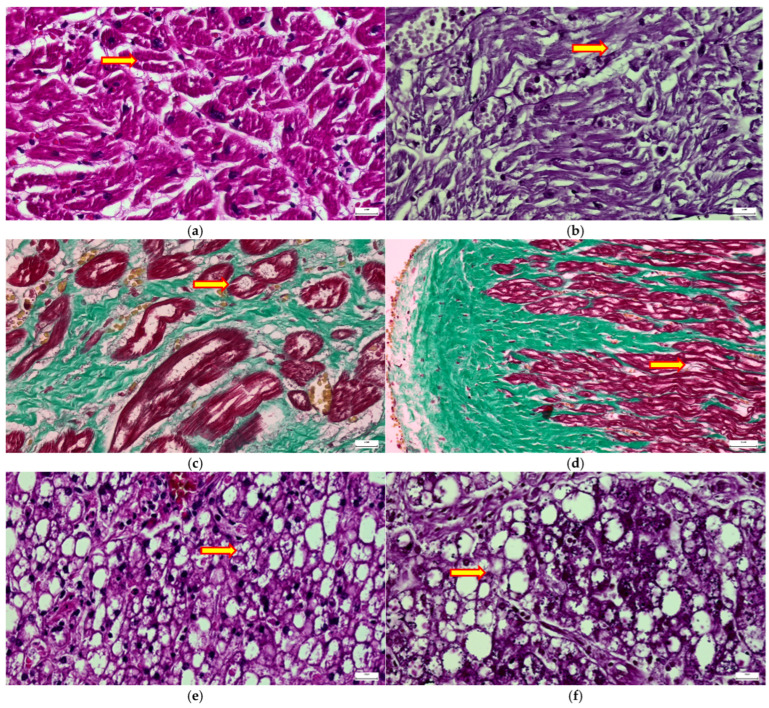

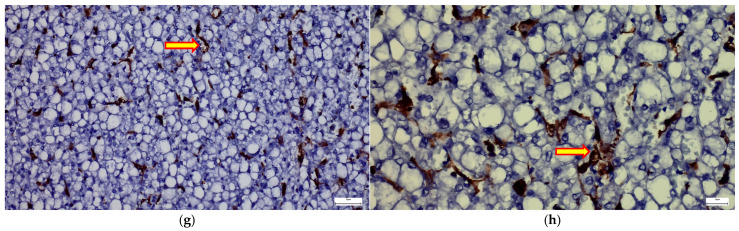
Systemic storage in cardiac and hepatic tissues. (i) cardiac findings: (**a**,**b**) cardiomyocytes in cross and longitudinal sections showing sarcoplasmic clearing and vacuolization (HE and PAS, 20 µm); (**c**,**d**) Masson’s trichrome stain highlighting extensive subendocardial and interstitial fibrosis (green) surrounding hypertrophied myocytes (red) (20 µm). (ii) Hepatic findings: (**e**,**f**) hepatocytes showing coarse cytoplasmic vacuolization and PAS-positive storage material (HE, 50 µm; PAS, 20 µm); (**g**,**h**) IHC for CD68 demonstrating a significantly increased population of hypertrophied Kupffer cells within the sinusoids (50 µm and 20 µm).

Myocardial fibres show cytoplasmic vacuolization and sarcoplasmic clearing consistent with lysosomal glycosaminoglycan accumulation within cardiomyocytes. Myocyte hypertrophy with architectural disarray, interstitial edema, and focal myofibrillar rarefaction are observed, reflecting progressive storage-related cellular dysfunction and early myocardial remodeling (Figure 6a,b).

Masson’s trichrome staining: increased subendocardial and interstitial and perivascular collagen deposition (green staining) highlights diffuse and patchy myocardial fibrosis, separating and entrapping cardiomyocytes (red) (Figure 6c,d).

These findings indicate chronic extracellular matrix remodeling and replacement fibrosis secondary to prolonged metabolic injury.

In the liver, hepatocytes and Kupffer cells were enlarged with clear vacuolated cytoplasm, corresponding to intralysosomal glycosaminoglycan accumulation (Figure 6e–h).

Histological analyses revealed diffuse parenchymal damage with extensive cytoplasmic clearing of hepatocytes, resulting in a foamy, microvacuolized appearance and slight architectural disorganization of the hepatic cords. Portal and sinusoidal regions showed swollen hepatocytes with prominent intracellular vacuoles consistent with lysosomal glycosaminoglycan accumulation, associated with sinusoidal narrowing and mild stromal reaction.

Ballooned hepatocytes were identified with coarse cytoplasmic vacuolization and displacement of nuclei, reflecting progressive lysosomal distension and metabolic storage injury, also extensive intracytoplasmic storage vacuoles within hepatocytes and Kupffer cells, producing marked cellular enlargement and parenchymal rarefaction.

Overall, these findings demonstrate diffuse visceral (hepatic) involvement in MPS III, characterized by intralysosomal glycosaminoglycan accumulation in hepatocytes and Kupffer cells, leading to hepatocellular swelling, sinusoidal compression, and progressive organ dysfunction.

Similar vacuolar changes were observed in renal tubular epithelial cells, pancreatic acinar cells showed similar microvacuolation without signs of chronic pancreatitis, and enteric ganglion cells.

**Figure 7:**
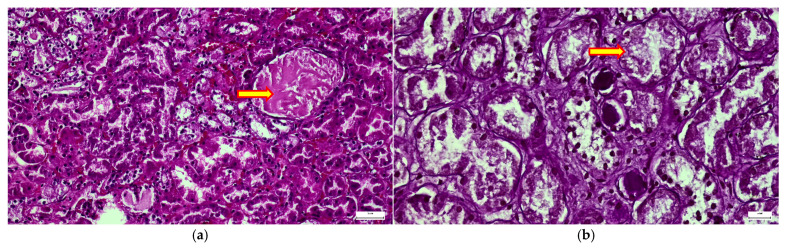

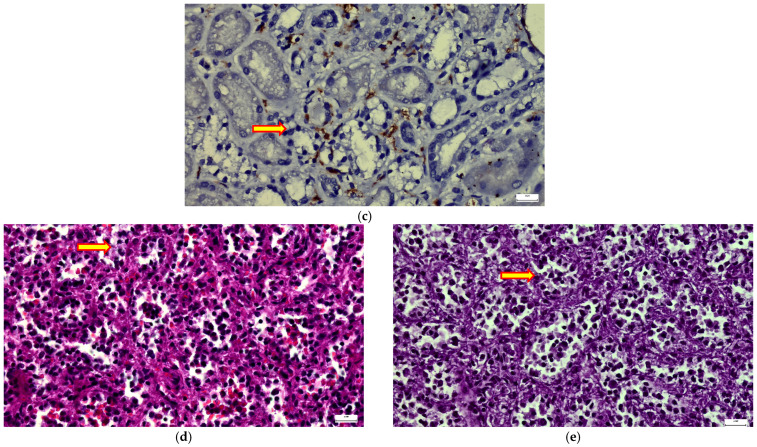

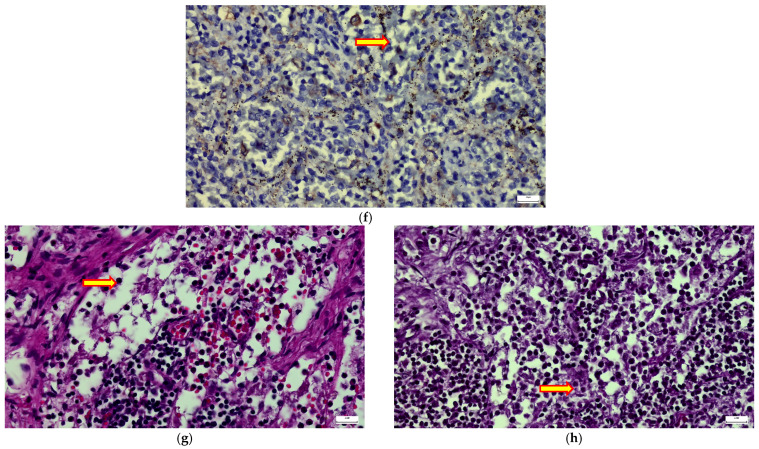

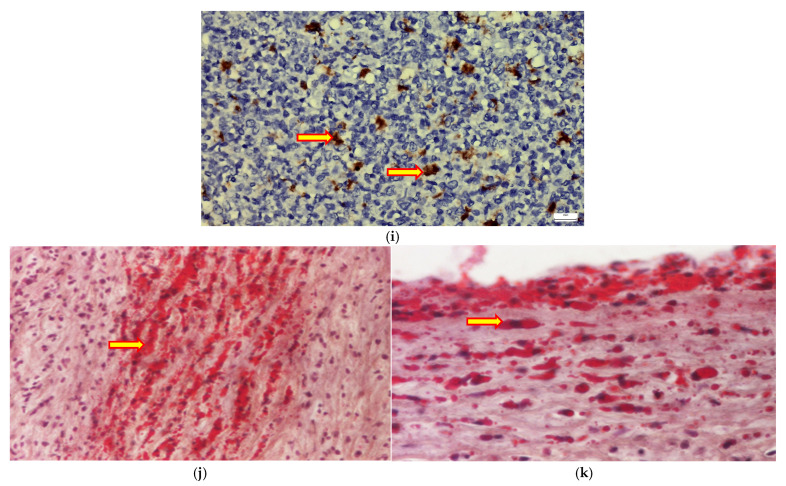
Multisystemic pathological mapping. (i) kidney: (**a**,**b**) tubular epithelial cells with prominent cytoplasmic vacuolization and PAS-positive inclusions (50 µm); (**c**) interstitial infiltration of CD68+ cells. (ii) spleen and lymph node: (**d**,**e**) splenic parenchyma showing foamy macrophages and PAS-positivity (50 µm); (**f**) marked histiocytosis in Billroth cords (CD68, 20 µm); (**g**–**i**) mesenteric lymph node architecture with extensive histiocytic vacuolization and CD68 reactivity (20 µm). (iii) aorta: (**j**,**k**) Scharlach stain confirming lipid-laden foamy macrophages (“fatty streaks”) within the subendothelial intimal layer (50 µm and 20 µm).

Microscopic analyses showed diffuse involvement of the renal parenchyma with cytoplasmic clearing and microvacuolar change affecting tubular epithelial cells, resulting in a pale, foamy appearance and mild architectural distortion (Figure 7a,b). Were also present swollen tubular epithelial cells with prominent intracytoplasmic vacuoles consistent with lysosomal glycosaminoglycan accumulation, associated with tubular luminal narrowing and interstitial congestion (Figure 7a), marked ballooning degeneration of epithelial cells with optically clear storage vacuoles and nuclear displacement, reflecting lysosomal distension and progressive metabolic storage injury (Figure 7b).

These findings support renal involvement in MPS III, characterized by intracellular lysosomal storage within tubular epithelium and interstitial cells (Figure 7c), contributing to tubular dysfunction and progressive parenchymal alteration.

The spleen and lymph nodes showed sinusoidal congestion and prominent histiocytosis with foamy macrophages (Figure 7d–i).

At the vascular level, the aortic intima revealed subendothelial aggregates of foamy macrophages arranged in bands and nests, positive for lipid staining (Scharlach stain), consistent with early atherogenic “fatty streak” lesions.

Microscopic examination reveals aggregates of foamy macrophages arranged in bands and nests within the subendothelial tissue. These cells show positive Scharlach staining, confirming intracellular lipid accumulation (Figure 7j,k). Representative microscopic fields at progressively higher magnifications illustrating the distribution and density of lipid-laden macrophages in the aortic intima.

The immunohistochemical staining for CD68 revealed an increased number of activated macrophages with cytoplasmic vacuolization, supporting the involvement of the reticuloendothelial system and the inflammatory component of systemic lysosomal storage disease, including the brain, most of them distributed in the perivascular areas (Figure 5c), the cerebellum (Figure 5e), the liver with a lot of Kupffer cells in the sinusoids (Figure 6g,h), the kidney (Figure 7c), the spleen (Figure 7f), and the lymph nodes (Figure 7i). Moreover, the number of CD68+ cells identified in our patient was significantly higher than in the healthy control patients analyzed (Figure 5f).

These findings confirm widespread lysosomal storage pathology with prominent multisystem involvement, accompanied by secondary vascular lipid accumulation.

## 4. Discussion

Mucopolysaccharidosis type III (MPS III) is clinically defined by its devastating neurodegenerative course, a feature that often overshadows its systemic impact. However, while the central nervous system remains the primary focus of clinical management, the underlying pathophysiology involves a silent, progressive accumulation of glycosaminoglycans across multiple organ systems [3,4].

As emerging therapies like enzyme replacement and gene editing begin to reshape the treatment landscape for lysosomal storage disorders, accurately mapping the systemic distribution of pathology becomes crucial.

The 15-year-old patient described here represents an advanced stage of MPS III. Her longevity provided a unique opportunity to document the extensive visceral pathology that often progresses silently beneath the dominant neurodegenerative phenotype.

The widespread cytoplasmic vacuolization observed across visceral organs reflects progressive intralysosomal glycosaminoglycan accumulation, a hallmark of lysosomal storage disorders [2,7,21,22], lesions that have also been reported in other studies [6,13]. Lysosomal distension disrupts intracellular metabolism, promotes chronic inflammation, and activates apoptotic pathways, ultimately leading to progressive tissue dysfunction [2,7]. Similar multisystem histopathological lesions have been reported in previous morphopathological studies of MPS patients [6,13].

While neurological deterioration defines the clinical course [8,10], visceral involvement plays a significant role in morbidity and mortality [9,14,23], with similar lesions also identified in our patient.

In our case, the pulmonary findings are consistent with terminal bronchopneumonia, a frequent complication in bedridden patients with advanced disease. The significant increase in lung weight and diffuse edema reflect the susceptibility of these patients to acute cardiorespiratory decompensation, often exacerbated by bulbar dysfunction and recurrent aspiration [17].

The heart showed cardiomegaly, chamber dilatation, and atrioventricular valvular thickening, while the myocardium appeared pale and soft, suggestive of myocardial degeneration associated with interstitial fibrosis. In mucopolysaccharidosis type III, cardiac involvement is commonly associated with glycosaminoglycan deposition in the valves and conduction system, contributing to cardiac dysfunction. These macroscopic findings support the occurrence of acute cardiorespiratory decompensation in the context of gradual multisystem involvement. Additionally, structural cardiac abnormalities, including myocardial degeneration and valvular thickening, further contributed to hemodynamic instability [12,18,19,21].

Furthermore, the observed cardiomegaly and valvular thickening suggest that chronic GAG deposition in the cardiovascular system contributes to hemodynamic instability and mortality [12,18,19,21]. The liver was moderately enlarged and exhibited chronic passive congestion, indicating systemic venous stasis.

Although a formal skeletal analysis was not conducted, the observed cranial vault thickening and severe osteoarticular contractures provide indirect evidence of the skeletal remodeling characteristic of dysostosis multiplex in MPS III.

Unlike MPS types I and II, where somatic manifestations are clinically conspicuous [1,15,16], systemic involvement in MPS III is often underestimated during life [4].

In these patients, the predominance of severe and rapidly progressive neurocognitive decline tends to overshadow extracerebral disease [8,9], diverting clinical attention away from visceral pathology. As a consequence, glycosaminoglycan accumulation within the liver, spleen, heart, kidneys, and reticuloendothelial tissues may progress silently, producing subtle or nonspecific manifestations that remain clinically underrecognized [13,14].

The severity of neurocognitive decline tends to divert clinical attention away from visceral pathology, allowing glycosaminoglycan accumulation within the liver, spleen, and kidneys to progress without specific recognition [13,14].

A comprehensive post mortem analysis evaluation, therefore, represents critical tool for accurately mapping the true extent of multisystem involvement, providing direct morphologic evidence of storage burden and organ-specific vulnerability [2,22]. Such post mortem findings not only enhance our understanding of disease pathogenesis and natural history but also have important translational implications. In particular, defining the tissue distribution and severity of storage pathology may inform emerging therapeutic strategies, including enzyme replacement therapy and gene therapy [23,24,25], by identifying target organs, therapeutic windows, and potential barriers to effective systemic delivery.

The histopathological severity documented here must be understood in the context of current therapeutic limitations.

While hematopoietic stem cell transplantation (HSCT) and enzyme replacement therapy (ERT) have successfully mitigated somatic symptoms in other MPS types (such as MPS I and MPS II) [26], their efficacy in Sanfilippo syndrome remains a subject of ongoing research.

Our patient followed the natural history of the disease; had systemic therapies been available and initiated early, the visceral glycosaminoglycan burden in the liver, spleen, and heart might have been reduced, although the CNS pathology, the primary driver of decline, would likely have remained unchanged due to the blood–brain barrier.

In addition, early lipid-rich intimal streaks with subendothelial foamy macrophages were observed in the aorta. Although MPS III is primarily characterized by glycosaminoglycan storage rather than primary lipid deposition, global lysosomal dysfunction may secondarily disturb cellular lipid turnover and promote foam cell formation.

Also, prolonged immobilization in advanced stages of the disease may promote chronic venous stasis, which likely contributed to the chronic passive hepatic congestion observed at post mortem analysis.

Similar mechanisms of secondary lipid accumulation and inflammatory activation have been described in lysosomal storage disorders, linking lysosomal dysfunction to altered lipid homeostasis and vascular pathology [27,28,29]. In the context of terminal-stage disease and palliative care, recognition of systemic involvement—especially gastrointestinal and cardiac manifestation—is essential for optimizing symptom control and improving quality of life in patients with advanced MPS III.

The histopathological findings in our case align with the limited number of previously reported autopsy studies in MPS III, which predominantly highlight neuronal ballooning and secondary gliosis. However, our study provides an expanded view of the somatic burden, particularly the early atherogenic changes in the aorta and the advanced valvular fibrosis, which are less frequently documented in detail.

The microscopic evaluation of the somatic and central nervous system tissues revealed significant PAS-positive inclusions within the cytoplasm of various cell types. However, it is important to acknowledge that PAS staining is not pathognomonic for glycosaminoglycans, as it primarily reacts with aldehyde groups formed by the oxidation of polysaccharides, glycoproteins, and glycolipids. While the intense PAS positivity observed in this case is highly suggestive of lysosomal storage, it lacks the specificity to definitively isolate heparan sulfate from other complex carbohydrates.

In the absence of more specific histochemical or immunohistochemical markers for GAG accumulation, our findings were interpreted through a multidisciplinary approach, correlating the morphological features with the patient’s confirmed genetic and biochemical diagnosis of MPS III A. This correlation supports the conclusion that the visualized storage material is consistent with the systemic lysosomal involvement characteristic of advanced-stage Sanfilippo syndrome.

While CD68 is a widely recognized marker for cells of the monocyte-macrophage lineage [30,31], we acknowledge the limitation of not employing more specific microglial markers, such as Iba1, or specialized somatic markers like Mac2. However, the robust CD68 positivity observed across both CNS and somatic tissues, when interpreted in conjunction with the characteristic ‘foamy’ cellular morphology, provides strong evidence of widespread macrophage and microglial activation.

In the context of MPS III A, CD68 expression is particularly informative, as it reflects not only the presence of these inflammatory cells but also the significant expansion of their lysosomal compartments due to heparan sulfate accumulation. A hallmark of the systemic pathology observed in our case was the presence of cells with a distinct ‘foamy’ or vacuolated appearance, particularly within the macrophage populations of the liver, spleen, and perivascular spaces of the brain. From a biochemical perspective, this morphological feature reflects the massive intralysosomal accumulation of heparan sulfate and associated secondary lipid metabolites, which are characteristic of advanced MPS III A.

Finally, we addressed the technical limitations of histopathological processing. While PAS staining and CD68 immunohistochemistry provide strong surrogate evidence of storage and macrophage activation, the “foamy” appearance of cells in FFPE sections represents the negative image of storage material extracted during dehydration. Despite these constraints, the correlation between clinical history, biochemical deficits, and characteristic morphological findings is sufficient to delineate the full spectrum of systemic involvement.

These findings corroborate the hypothesis that the pathology of Sanfilippo syndrome type A extends far beyond primary neurological damage, involving a complex, multi-organ inflammatory response driven by lysosomal dysfunction.

## 5. Conclusions

Mucopolysaccharidosis type III is best understood as a systemic and progressive lysosomal storage disorder with widespread multisystem involvement, rather than an exclusively neurodegenerative disease. Recognition of these systemic alterations supports the need for a multidisciplinary approach, extending beyond purely neurological management, even in advanced stages of the disease. The present case of a 15-year-old patient of relatively advanced age for the severe clinical spectrum of MPS III provided a unique opportunity to document the full morphological extent of the disease. Survival into adolescence, likely facilitated by comprehensive palliative care, allowed for the observation of extensive intralysosomal glycosaminoglycan accumulation across the central nervous system, cardiovascular structures, and visceral organs.

The presence of early lipid-rich aortic intimal streaks suggests secondary vascular lipid accumulation related to global lysosomal dysfunction and chronic inflammation. While not disease-specific, these changes, captured at an advanced stage of disease progression, expand the known morphological spectrum of MPS III.

Comprehensive clinicopathological correlation through post mortem analysis remains essential for accurately delineating disease extent and clarifying mechanisms of terminal decompensation. Such findings are crucial for informing future systemic therapeutic strategies that must target both neurological and extracerebral pathology to address the total disease burden.

## Data Availability

The original contributions presented in this study are included in the article. Further inquiries can be directed to the corresponding author.
